# Banquet in Honor of Dr. S. B. Palmer, of Syracuse, N. Y.

**Published:** 1902-12

**Authors:** G. Alden Mills

**Affiliations:** New York City


### BANQUET IN HONOR OF DR. S. B. PALMER, OF SYRACUSE, N. Y.

NEW YORK CITY, October 27, 1902.

TO THE EDITOR:

SIR,—Nothing that we have attended in many a day has been more enjoyable than that of a party of some thirty-five who sought to do honor to one of our most worthy members, Dr. S. B. Palmer, of Syracuse. The table was set at the palatial New York Athletic Club, and this with abundant satisfaction. Planked shad figured bountifully and enticingly. Everything was in the simplest form, and presided over by Dr. A. H. Brockway, who of himself is pure and simple. The list of speakers speaks for itself. Everything breathed a tender and truth-saying regard for our guest. Nothing spectacular save the speech of Dr. Parmly Brown, son of the noted late Salymon Brown. He did not play leap-frog, but he did—the frog in his throat, so much so it was whispered to us he has coming on a case of vocal paralysis; but to the astonishment of all, he, by a strangulating cough, brought out (or seemed to) a veritable live dangling bull-frog from his mouth. Did it create a guffaw? Of course it did; this ending by a dramatic production of Shakespeare in marked effect, and closing with a beautiful poem by his father, nicely given. One speaker referred to another gathering of dentists that had sought to crown a Prince of Siam that eve, at the Lotus Club, but *we* were crowning a *real* prince among our own craft.

Reference was made, by way of illustration, to the keen disappointment of the late Dr. Atkinson's visit to Europe, he not receiving the attentive appreciation that he thought due, as he told a friend that they dined him and wined him, and after that they had no more use for him (Dr. Atkinson did not see that they were following "the customs of the country"). Not so is it to be with Dr. Palmer; we hope to use him until the end of his career. Drs. Brockway, Curtis, and Hodson, all formerly of Syracuse, vied with each other for a tender and emphatic expression of good-will for the doctor. He had been in their early days truly, if not a "cherishing mother," certainly a "cherishing father," to Dr. Curtis a means of the possibility of his larger education, which has notably brought to him a high position in his surgical department, which will not fail to do honor to him later. To Dr. Curtis is most largely due this delightful pastime and fraternal intercourse in the ripening days of our friend and co-worker.

We cannot fail to notice with tender regard one of the letters of choice wording from our greatly appreciated co-laborer, Professor Truman, informing us of his severe sickness, preventing his anticipated part in doing honor to Dr. Palmer, for whom he expressed his decided friendship and appreciation. Much solicitation was manifested for our worthy editor and friend and co-laborer, trusting that he may have a speedy return to his accustomed health. Dr. Truman sent the following sentiment:

> "'This above all: to thine own self be true, . . .
> Thou canst not then be false to any man.'

"The man who selfishly regards himself as superior to all others will drive all others from his realm of thought. We honor Dr. Palmer because he exemplifies in his own person consistent ideals, unselfish devotion to principles, as he understood them, leaving the final solution of the problem he sought to cover to the infallible test of time."

Dr. Kingsley referred to the fact that out of forty "Patriarchs" that were given a banquet some twelve years ago, they having passed their fiftieth year of practice, only two of them were now living,—Drs. John B. Rich, now nearly ninety-two years old, and William B. Hurd, eighty-three, the latter having held a reception that afternoon, bringing together, as he expressed it amusingly, the greatest gathering of antiquated bric-à-brac he had ever met, one of them an even one hundred years old. It was expressed as quite a marvel to note with what vigor of voice Dr. Rich spoke in regard to Dr. Palmer. Even at his age he is actively conducting successfully a School of Physical Culture, being a lifelong expert in this connection, which doubtless has much to do with his longevity. A number of dentists have been and are being largely benefited by this culture.

Dr. Palmer announced in his closing remarks a *prediction*,—viz., there is to be a *new* physiology taught that will be an astonishment to the scientific world. Following this, Dr. Palmer gave an outline of his views in this connection, which we give as he has put it into shape for publication, and we predict it will be read with much interest and curiosity.[1] Should this prediction ultimately prove a fact, this dinner-party will be recalled as an eventful one in history. All honor and longer life to our esteemed co-laborer, Dr. Palmer.

G. Alden Mills.

[1] For Dr. Palmer's paper, see page 897.

